# The Prevalence and Severity of Sick Leave due to Low Back Disorders among Workers in Slovenia: Analysis of National Data across Gender, Age and Classification of Economic Activities

**DOI:** 10.3390/ijerph19010131

**Published:** 2021-12-23

**Authors:** Dorjana Zerbo Šporin, Žiga Kozinc, Ticijana Prijon, Nejc Šarabon

**Affiliations:** 1 Faculty of Health Sciences, University of Primorska, Polje 42, SI-6310 Izola, Slovenia; dorjana.zerbosporin@fvz.upr.si (D.Z.Š.); ziga.kozinc@fvz.upr.si (Ž.K.); 2 Andrej Marušič Institute, University of Primorska, Muzejski trg 2, SI-6000 Koper, Slovenia; 3 National Institute of Public Health, Trubarjeva 2, SI-1000 Ljubljana, Slovenia; Ticijana.Prijon@nijz.si; 4 Human Health Department, InnoRenew CoE, Livade 6, SI-6310 Izola, Slovenia; 5 Laboratory for Motor Control and Motor Behavior, S2P, Science to Practice, Ltd., Tehnološki Park 19, SI-1000 Ljubljana, Slovenia

**Keywords:** musculoskeletal pain, absenteeism, epidemiology, workplace, back pain

## Abstract

Musculoskeletal disorders are the most common work-related health problems. As low back disorders (LBD) are the most problematic, the aim of this study was to provide an in-depth analysis of the nationwide data on sick leaves due to work-related LBDs among workers in Slovenia in 2015–2019 by gender, age and various economic activities (NACE Rev 2 classification). We retrospectively analyzed the Slovene national data for sick leave (SL) rates due to the LBDs between 2015 and 2019. The analyzed SL outcomes were (i) index of temporary disability as a diagnosis-specific loss of calendar days (all calendar days except Sundays) per employee, (ii) frequency of spells as the number of SL cases per 100 employees in one year and (iii) severity as the average duration of one absence from work due to a health condition. A high prevalence of sick leaves due to LBDs in Slovenia was present among young male workers in “mining and quarrying”. In the next age group (20.0–44.9 years), LBD is most frequent in “water supply; sewerage, waste management and remediation activities”. Particular attention should be paid to ‘’agriculture, forestry and fishing’’ which shows a large average sick leave duration and probably a more demanding course of LBDs.

## 1. Introduction

Musculoskeletal disorders (MSDs) are the most common work-related health problem in the European Union (EU) [1]. According to the Centre for Disease Control and Prevention, work-related MSDs are injuries or disorders of the muscles, nerves, tendons, joints, cartilage and spinal discs in which (i) the work environment and performance of work contribute significantly to the condition and/or (ii) the condition is made worse or persists longer due to work conditions [2]. Various factors may contribute to MSDs, such as physical, organizational, psychosocial, sociodemographic and individual factors [1]. Psychosocial risks, especially in combination with physical risks, may cause or aggravate MSDs [3]. 

In 2015, three out of five workers in the EU-28 reported MSDs. Sixty-three percent of workers in Slovenia reported that they suffered from one or more MSDs in the past 12 months [1]. While the prevalence of musculoskeletal conditions increases with age, younger people are also affected [4]. In addition to the normal degenerative aging process, poor work environment and performance of work contribute significantly to the condition [2]. MSDs concern workers in all sectors and occupations, but a high prevalence of self-reported MSDs is most often reported by workers employed in the sector of construction, water supply and agriculture, forestry and fishing, and is above average among human health and social work activities [1]. MSDs are one of the most significant diseases contributing to work disability [5] and they lead to high costs to enterprises and society [1].

With an aim to reduce the incidence of MSDs and thus the economic and social consequence of MSDs and according to the objectives of the EU Strategic Framework of Health and Safety at Work 2014–2020 [6], in 2020, the project “Promotion of activities to prevent musculoskeletal disorders and psychosocial risk in the workplace (2020–2021)” (OP20.05955) in Slovenia was established. This national project that is jointly run by the National Institute of Public Health (lead partner) and the University of Primorska, Faculty of Health Science (project partner) implements a holistic approach to preventing and managing the incidence of work-related MSDs and psychosocial risk using a range of systematic activities: workplace ergonomics, physical activity and psychological interventions, all included in a friendly e-tool (www.pkmo.si, accessed on 11 November 2021).

In the project’s first phase, a retrospective analysis of sick leave (SL) incidence due to the most common work-related MSDs was carried out based on health statistics. In the period 2015–2019, the average loss of calendar days due to work-related MSDs was 2.3 per employee, with eight annual cases per 100 employees. The average duration of one MSDs related SL was 33.5 days. Within MSDs, the highest percentage of SL was detected for work-related low back disorders (LBDs) (56.6% of all lost working days). In the observed period, the average loss of calendar days due to LBDs was 1.3 per employee with four annual cases per 100 employees. The average duration of one LBD-related sick leave (SL) was 30.7 days. The loss of calendar days per employee was higher in females than in males (1.53 and 1.38, respectively) and reached its peak (2.7) in the 45–64 age group [7,8].

LBDs are common in all age groups [9], with the lifetime prevalence as high as 75 to 84% [10] and point prevalence ranging from 1.4 to 20.0% across populations and geographical locations [11]. Risk factors for work-related musculoskeletal disorders are psychosocial, individual and biomechanical [12]. Although LBDs may occur as a result of specific pathologies, such as damage to the intervertebral disc, damage to the intervertebral joints or nerve impingements [13], in most cases, the underlying cause remains unknown [14,15]. Therefore, the prevention and treatment of LBDs remain a huge challenge [16,17]. Chronic pain syndromes have a severe negative impact on the patients and people around them, affecting their social life and overall life quality [18,19]. Medical conditions also represent a large burden for healthcare systems. For instance, reports from Sweden estimated the annual costs due to LBDs at 740 million euros [20]. Studies from around the world have reported that the majority of the costs related to LBDs are indirect (i.e., due to sick leaves and reduced work ability) [21,22]. In the working-age population, between 20 and 40% of persons suffer from LBDs annually [23]. Effective interventions for the prevention and rehabilitation of LBDs would likely unload the public healthcare systems across the world, in addition to helping the patients directly. Several studies have already shown a great potential of physical activity and exercise in the treatment and prevention of LBDs [24,25,26]. In addition to exercise, educating workers has also been suggested as an effective approach to preventing LBDs [27]. However, given the abovementioned impacts of LBDs on the individual, their family and society, further work needs to be done to prevent and treat LBDs more effectively. 

As LBDs are evidently the most common work-related MSDs, the aim of this study was to provide an in-depth analysis of the nationwide data on sick leaves due to work-related LBDs among workers in Slovenia in 2015–2019 by gender, age and various economic activities. The scope was to identify and latterly present the standing out data that could be useful to decision-making in developing LBDs control strategies in Slovenia and globally. 

## 2. Materials and Methods

### 2.1. Study Population and Data Collection

We retrospectively analyzed the Slovene national data for sick leave (SL) rates due to the most common work-related low back disorders (LBDs) in relation to gender, four age groups (15–19.9 years, 20–44.9 years, 45–64.9 years and 65+ years) and following the incidence of LBDs across different economic activities according to the NACE Rev 2 classification [28]. Table 1 shows the study sample (i.e., the number of employed personnel across calendar years for both genders). In Table 2, the data is further broken down by age groups, gender and economic activities. Persons from the group over 65 years in our study are still employed. The right to an old age pension in Slovenia is regulated by the Pension and Disability Insurance Act. The law lays down the conditions under which a person who satisfies them joins compulsory pension. In order to qualify for an old age pension, one must meet the following general conditions: (i) 60 years of age and 40 years of pensionable service without purchase or (ii) 65 years of age and a 15-year insurance period [29]. It is not mandatory that the employee must also terminate the employment relationship under these conditions. An employment contract may be concluded by persons who have reached the age of 15 [30]. Within young workers (15.0–19.9 years and 20.0–44.9 years), persons performing student work are compulsorily insured [29].

Data regarding SL represent an important source of information on the health status of the working population. The purpose of data collection is to monitor and analyze the pattern of sick leave or temporary absence from work as a result of disease, injury or other health conditions. The National Institute for Public Health (NIJZ) is the institution responsible for collecting official data on SL in the working population in which employed and self-employed persons working in Slovenia are included (Table 1). Data collection has a legal basis in the Health Care Databases Act (ZZPPZ—Ur. l. RS 65/00, database NIJZ3) and Personal Data Protection act (ZVOP-1—Ur. l. RS 94/07), which gives the basis for further processing of data for scientific research or historical or statistical purposes. The source of data is the Certificate of justified abstinence from work due to health conditions (eBOL). The data is obtained from health care providers regarding the decisions of personal general practitioners who ensure the need of absence from work. After the 30th day of SL, the decision must be confirmed by an appointed doctor from the Health Insurance Institute of Slovenia [31]. All data were anonymous at all stages of the study.

### 2.2. Classification of Economic Activities

The study population was segregated according to the NACE Rev 2—Statistical classification of economic activities in the European Community [28]. According to this classification, the activities are separated into 21 categories: (A) Agriculture, forestry and fishing, (B) Mining and quarrying, (C) Manufacturing, (D) Electricity, gas, steam and air conditioning supply, (E) Water supply; sewerage, waste management and remediation activities, (F) Construction, (G) Wholesale and retail trade; repair of motor vehicles and motorcycles, (H) Transportation and storage, (I) Accommodation and food service activities, (J) Information and communication, (K) Financial and insurance activities, (L) Real estate activities, (M) Professional, scientific and technical activities, (N) Administrative and support service activities, (O) Public administration and defense; compulsory social security, (P) Education, (Q) Human health and social work activities, (R) Arts, entertainment and recreation, (S) Other service activities, (T) Activities of households as employers; undifferentiated goods- and services-producing activities of households for own use and (U) Activities of extraterritorial organizations and bodies.

### 2.3. Outcome Measures

For the analysis, we received anonymous data as numbers representing SL rates by gender, age and NACE Rev 2 classification of economic activities. The analyzed SL rates were: (i) index of temporary disability as a diagnosis-specific loss of calendar days (all calendar days except Sundays) per employee, (ii) frequency of spells as the number of SL cases per 100 employees in one year and (iii) severity as the average duration of one absence from work due to a health condition. The number of SL cases is considered to be the number of completed SL duo to LBDs in a calendar year (1 January–31 December) regardless of when SL started [31,32]. The average values for SL rates from 2015 till 2019 were used for analysis.

According to the MKB-10-AM, diagnoses for the most common work-related LBDs are classified as follows: (i) lumbar spine intervertebral disc defects (M51.0–M51.9), (ii) ischialgia (M54.3), (iii) lumboischialgia (M54.4) and (iv) lumbalgia (M54.5) [8].

## 3. Results

The overall results for days of absence, number of cases and average case duration because of sick leave due to low back disorders are presented separated by age, gender and NACE Rev 2 classification of economic activities, in Table S1. For clarity, we separated the graphical results by age groups.

**Figure 1:**
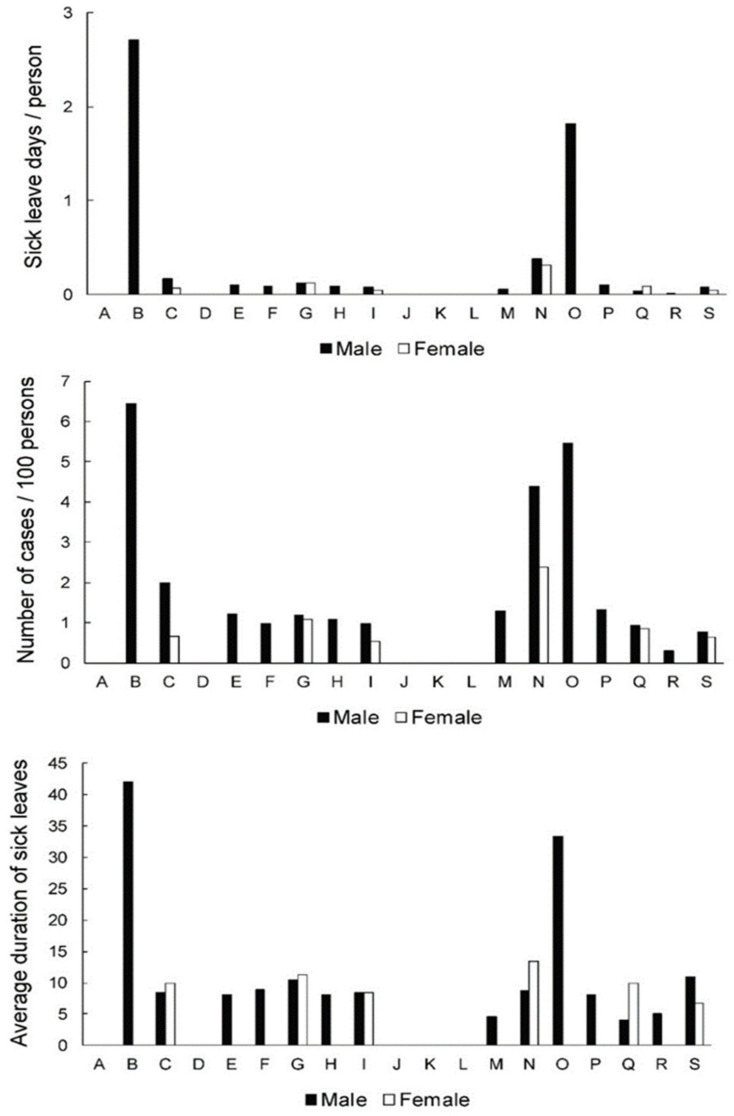
Sick leave days per person (index of temporary disability,) frequency of cases and severity (average case duration) of sick leave across genders and activities in 15.0–19.9-year-old group. A—agriculture, forestry and fishing; B—mining and quarrying; C—manufacturing; D —electricity, gas, steam, air cond. supply; E—water suppl; sewer., wst. manag., remed. act; F—construction; G—wholesale, retail; repair of mot. vehicles; H—transportation and storage; I—accommodation and food ser. activities; J—information and communication; K—financial and insurance activities; L—real estate activities; M—professional, scientific, technical act.; N—administrative and support service act; O—public admin, defense; compulsory soc. sec.; P—education; Q—human health and social work; activities; R—arts, entertainment and recreation; S—other service activities.

Figure 1 presents the data for the 15–19.9 age group. Regarding the loss of calendar days, males working in “mining and quarrying” (B) and “public administration and defence; compulsory social security” (O) activities presented by far the largest numbers (2.70 and 1.81 days per individual, respectively). For the males in other activities, as well as females in all activities, much lower values were documented (all < 0.5). The males in the abovementioned activities (B and O) also had the highest SL frequency (6.5 and 5.5 per 100 persons, respectively). Additionally, both males (4.4) and females (2.4) in “administrative and support service activities (N)” also presented with high numbers of annual cases of sick leaves. In the remaining activities, the number of annual cases was ≤2.0. Finally, the average severity of the sick leave was also the highest in the previously mentioned activities (B and O) in males (42.0 and 33.3, respectively). For the remaining groups that had documented cases, the severity of sick leaves was 4.5–11.3 days. Note that some activities did not have any documented cases in this age group (refer to the middle section of Figure 1, depicting the number of cases, and Table S1 for details). 

**Figure 2:**
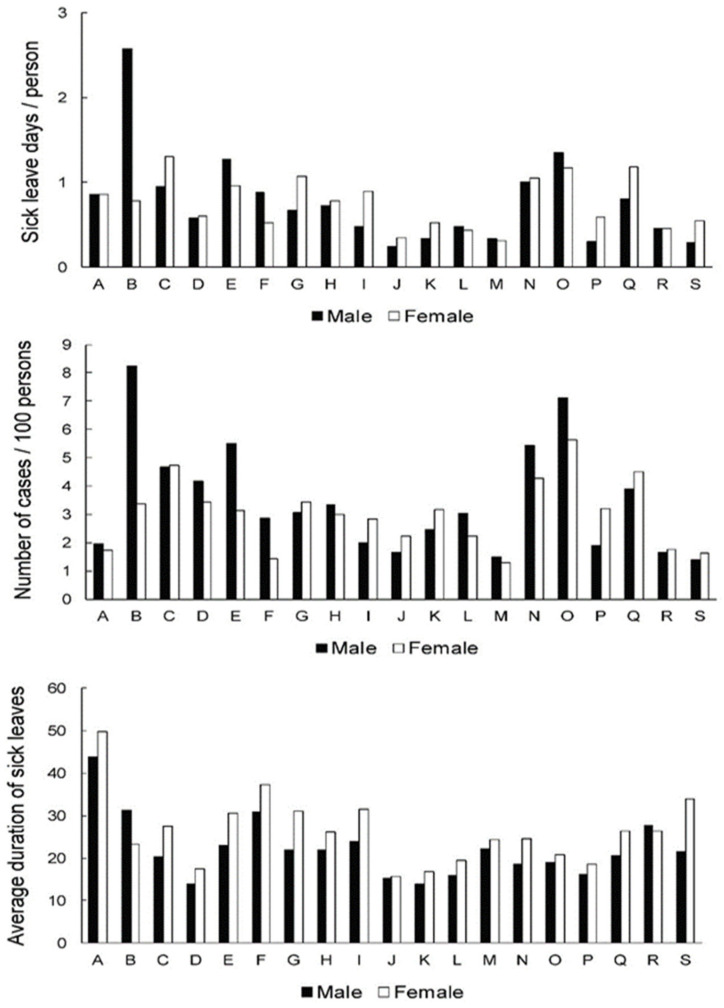
Sick leave days per person (index of temporary disability,) frequency of cases and severity (average case duration) of sick leave across genders and activities in 20.0–44.9-year-old group. A—agriculture, forestry and fishing; B—mining and quarrying; C—manufacturing; D —electricity, gas, steam, air cond. supply; E—water suppl; sewer., wst. manag., remed. act; F—construction; G—wholesale, retail; repair of mot. vehicles; H—transportation and storage; I—accommodation and food ser. activities; J—information and communication; K—financial and insurance activities; L—real estate activities; M—professional, scientific, technical act.; N—administrative and support service act; O—public admin, defense; compulsory soc. sec.; P—education; Q—human health and social work; activities; R—arts, entertainment and recreation; S—other service activities.

Figure 2 presents the data for the 20–44.9 age group. Regarding the sick leave days, “the male “mining and quarrying” (B) group stood out, with 2.57 days per person. The results for the remaining activities ranged from 0.25 to 1.35 in males and from 0.35 to 1.30 in females. It seems that the groups that had no cases in the 15.0–19.9 group tended to be less affected in this group as well (i.e., activities “agriculture, forestry and fishing” (A), “electricity, gas, steam and air conditioning supply” (D), ‘’information and communication’’ (J), “financial and insurance activities” and (K) and “real estate activities” (L)). Males in “mining and quarrying” (B) again had the highest number of cases (8.2 cases per 100 persons). Moreover, both genders in “administrative and support service activities” (N) and “public administration and defence; compulsory social security” (O) also had high numbers of cases (5.5 and 7.1 for males, and 4.3 and 5.6 for females). Another group with a high number of cases were males in “water supply; sewerage, waste management and remediation activities” (E), with 5.5 cases per 100 persons. Interestingly, the average duration of the sick leave was the highest in ‘’agriculture, forestry and fishing’’ (A) (43.8 days for males and 49.9 days for females). Females tended to be on sick leave for a slightly longer time in most groups. No other group stood out with a particularly low or high average duration of sick leave compared to others, although the range of data was fairly wide (13.8–31.2 days for males and 15.7–37.2 days for females).

**Figure 3:**
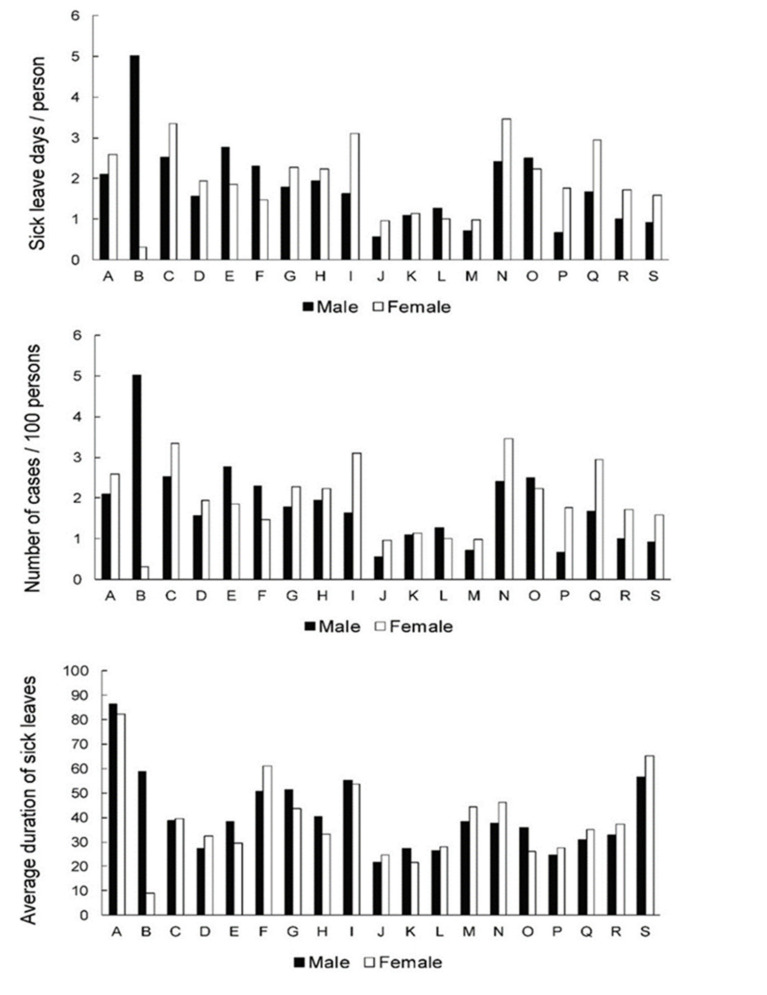
Sick leave days per person (index of temporary disability,) frequency of cases and severity (average case duration) of sick leave across genders and activities in 45.0–64.9-year-old group. A—agriculture, forestry and fishing; B—mining and quarrying; C—manufacturing; D —electricity, gas, steam, air cond. supply; E—water suppl; sewer., wst. manag., remed. act; F—construction; G—wholesale, retail; repair of mot. vehicles; H—transportation and storage; I—accommodation and food ser. activities; J—information and communication; K—financial and insurance activities; L—real estate activities; M—professional, scientific, technical act.; N—administrative and support service act; O—public admin, defense; compulsory soc. sec.; P—education; Q—human health and social work; activities; R—arts, entertainment and recreation; S—other service activities.

Figure 3 presents the data for the 45.0–64.9 age group. As in the two previous age groups, males in “mining and quarrying” (B) had the most sick leave days (5.0 days per person). However, contrary to the previous age groups, the next highest scores were present in 4 groups for females: “manufacturing” (C), “accommodation and food service activities” (I), “administrative and support service activities” (N) and “human health and social work activities” (Q) (3.3, 3.1, 3.5 and 2.9 days per person, respectively). Several groups presented with a high number of cases, often exceeding 5.0 per 100 persons. Three groups presented with a notably lower number of cases in both genders: “agriculture, forestry and fishing” (A) (2.4 and 3.1 cases for males and females, respectively), “professional, scientific and technical activities” (M) (1.8 and 2.1 cases for males and females, respectively) and “other service activities” (S) (1.6 and 2.4 cases for males and females, respectively). As in the previous age group, the average duration of sick leave was the highest in “agriculture, forestry and fishing” (A) (86.3 days for males and 82.4 days for females). While the average duration of sick leave was fairly similar across other groups, somewhat higher numbers were documented for both genders in “construction” (F) (50.6 days for males, 61.2 days for females), “accommodation and food service activities” (I) (55.2 days for males, 53.6 days for females), “other service activities” (S) (56.5 days for males, 65.2 days for females) as well as in “mining and quarrying” (B) for males (58.1 days).

**Figure 4:**
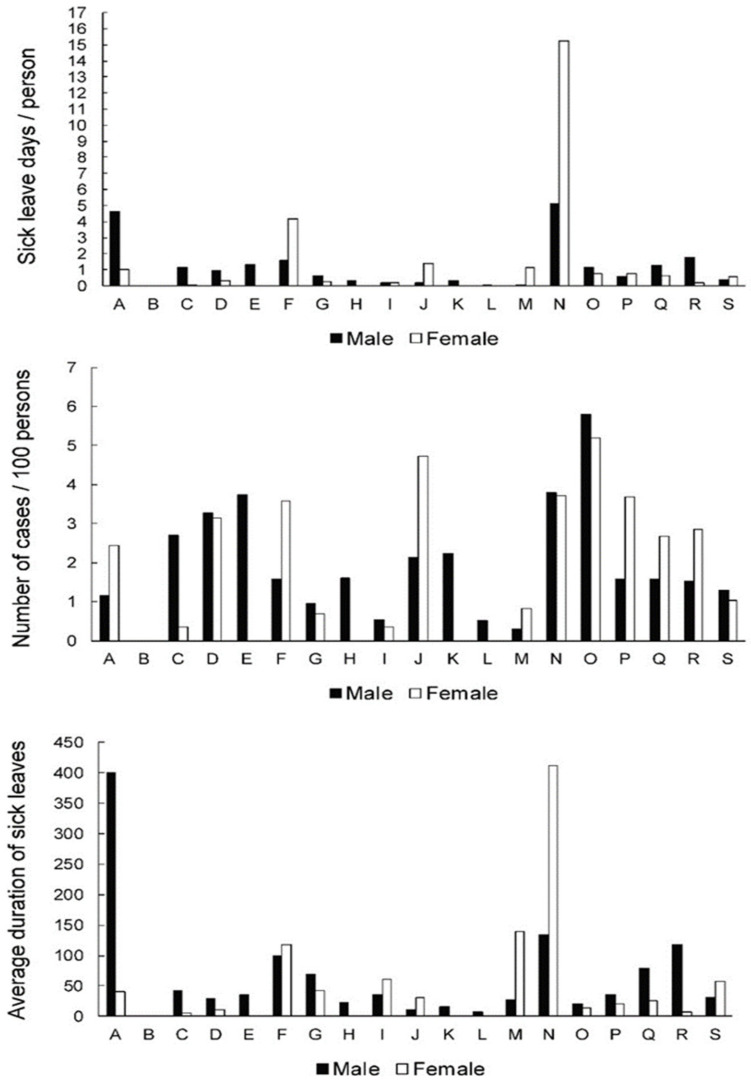
Sick leave days per person (index of temporary disability,) frequency of cases and severity (average case duration) of sick leave across genders and activities in >65-year-old group. A—agriculture, forestry and fishing; B—mining and quarrying; C—manufacturing; D —electricity, gas, steam, air cond. supply; E—water suppl; sewer., wst. manag., remed. act; F—construction; G—wholesale, retail; repair of mot. vehicles; H—transportation and storage; I—accommodation and food ser. activities; J—information and communication; K—financial and insurance activities; L—real estate activities; M—professional, scientific, technical act.; N—administrative and support service act; O—public admin, defense; compulsory soc. sec.; P—education; Q—human health and social work; activities; R—arts, entertainment and recreation; S—other service activities.

Figure 4 presents the data for the >65 age group. In this group, the females in “administrative and support service activities” (N) presented with particularly high numbers of sick leave days (15.2 days per person). In addition, high numbers were observed for females in “construction” (F) (4.2 days per person) and the males in “administrative and support service activities” (N) and “agriculture, forestry and fishing” (A) (5.1 and 4.6 days per person, respectively). The frequency of cases was more comparable across activities, with the highest number of cases for both genders reported for “public administration and defence; compulsory social security” (O) (5.8 and 5.2 cases per 100 persons for males and females, respectively). An extremely high average of severity of sick leave was observed for males in “agriculture, forestry and fishing” (A) and females in “administrative and support service activities” (N) (401.0 and 411.5 days, respectively).

**Figure 5:**
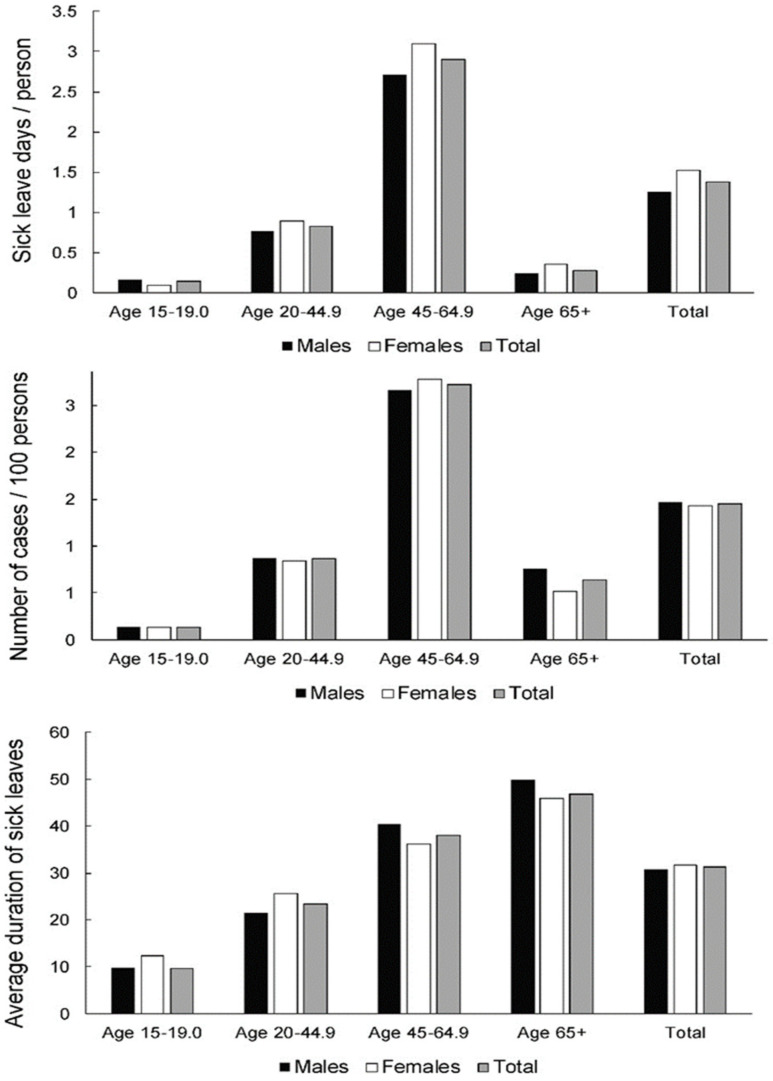
Sick leave days per person (index of temporary disability,) frequency of cases and severity (average case duration) of sick leave across genders and age groups across all economic activities.

Figure 5 presents the cumulative data across all economic activities. Sick leave days and frequency of cases seem to show very similar patterns, with the 45.0–64.9 years old group being affected the most and the 15.0–19.0 group being affected the least. However, in terms of the duration of sick leave, there is a clear increasing trend with age. There seem to be very few differences between sexes. Males in the older two groups tend to have slightly longer average sick leave, while the opposite is the case in the younger two groups.

## 4. Discussion

Musculoskeletal disorders are the most common work-related health problems in the EU [1] and are one of the most significant diseases contributing to work disability [5]. In addition to aging [4], poor working environments are also responsible for the occurrence of MSDs [2]. Low back disorders are the most predominant MSDs over the world [23,33] and in Slovenia as well [7,8]. The aim of this study was to provide an in-depth analysis of the nationwide data on sick leave rates due to work-related LBDs in 2015–2019 by gender, age and various economic activities. 

Among young employees (15.0–19.9 years), males working in “mining and quarrying” and “public administration and defence; compulsory social security” activities presented by far the largest numbers for LBDs specific loss of calendar days per employee and the number of cases per 100 employees (Figure 1). These activities also show the longest average duration of LBD-related sick leave. This is understandable, given that in other activities of this age group, there are little or no cases of sick leave. However, it should be pointed out that the number of young male workers in “mining and quarrying” and “public administration and defence; compulsory social security” is very low (Table 2); therefore, the sick leave duration in these activities is largely determined by a small number of individual cases. “Public administration and defence; compulsory social security” includes activities of a governmental nature, normally carried out by the public administration. It covers legislative activities, taxation, national defense, public order and safety, immigration services, foreign affairs and the administration of government programs [28]. In this activity, several physical and psychosocial risk factors for MSDs have been reported [34].

According to the larger number of young employees in “administrative and support service activities” compared to, for example, “mining and quarrying” (Table 2), the presented higher frequency of cases (Figure 1 is somewhat contradictory regarding LBDs cases among young workers in this activity. “Administrative and support services” are covering activities that support general business [28]. According to LBDs in sub-activities, we can find those that involve physically demanding work, e.g., cleaning activities, those linked to sedentary working environment e.g., office administrative support and those which could be connected to an elevated psychosocial risk e.g., travel agency and private security activities. Interestingly, “administrative and support service activities” remain endangered for LBDs in all age groups (Figure 2, Figure 3 and Figure 4), but the prevalence and severity of sick leave seem to be increasing in females during aging. We advise finding out what are the risk factors for LBDs in “administrative and support service activities” starting from the very beginning in young employees.

Returning to “mining and quarrying,” accompanied by unfavorable sick leave rates due to LBDs in males for almost all age groups, we might assume that demanding physical work increases the risk for LBDs in this activity. The high incidence of low back pain among miners is believed to be due to frequent awkward postures, manual handling and other heavy work and exposure to vibrations in the working environment [35,36]. Because of the demanding physical work, miners in Slovenia are included in occupational insurance. Under the terms of Pension and Disability Insurance Act ZPIZ-2 legislation [29], occupational insurance includes insured persons who perform particularly difficult and unhealthy work and persons who perform work that cannot be successfully performed professionally after a certain age. On the basis of collected funds, those insured acquire the right to count the insurance period with an added period, meaning to acquire the right of earlier retirement. Therefore, at age 65+ we can find no sick leave cases (Figure 4) in “mining and quarrying,” which means that sick leave cases are not documented but LBDs in elderly miners probably persist. 

Compared to the younger (15.0–19.0 age) group, sick leave rates for LBDs in workers from the 20.0–44.9 age group are expressed in all NACE Rev2 economic activities (Figure 2). In addition to the previously mentioned activities (that have evidently high SL rates for LBDs in almost all age groups), an increased frequency of cases for males in “water supply; sewerage, waste management and remediation activities” occurs in the 20.0–44.9 age group. Studies related to waste management reported forced postures in waste collection movements, mainly in lifting and unloading tasks [37]. In this age group, as in 45.0–64.9 years old (Figure 3), we found interesting the large increase in the average duration of sick leave due to LBDs in both genders for ‘’agriculture, forestry and fishing’’ in contrast to the relatively low number of recorded LBDs cases in this activity (Figure 2). From the Republic of Slovenia statistical office (SiStat) data for December 2019 [38], it is evident that a large proportion (82.3%) of the 24.364 employees in ‘’agriculture, forestry and fishing’’ are self-employed. It could be assumed that due to the socio-economic circumstances arising from their form of employment, fewer workers utilize sick leave for LBDs or only when the disease is already advanced, which is reflected in a low frequency but a high duration of sick leave (Figure 2). A similar deviation (low frequency, but long duration of sick leaves) (Figure 2) was found in “construction,” a primarily physically demanding working environment; however, a relatively lower percentage (37%) of people were self-employed in this activity [38]. Workers in “agriculture, forestry, and fishing” are exposed to MSDs risk factors such as repetitive movements, lifts, vibrations and awkward postures [39].

Female workers in the 20.0–44.9 age group with LBDs mostly remain on sick leave longer than males (Figure 2). This observation could reflect a more demanding course of the disease in female workers at this age. Female gender was identified as a predictor for longer sick leave in acute low back pain [40]. This was not the case in our study at age 45.0–64.9 years in “manufacturing” and in “human health and social work activities,” where an increase in the frequency of sick leaves due to LBDs occurs mainly among females, but the average duration of sick leaves remains comparable between genders (Figure 3). It seems that 45.0–64.9-year-old females in these activities are more susceptible to LBDs than age-matched males but do not show a more severe course of the disease. In “human health and social work activities,” risk factors for LBDs could be linked to physically demanding work, e.g., handling patients and psychosocial risk factors, which are both reported frequently in the healthcare sector [41]. In “manufacturing,“ forceful physical activity and material handling tasks represent the main risk [42].

The average sick leave episode duration due to LBDs increases for both genders in this group as well as in “accommodation and food service activities and “other service activities”. “Accommodation and food service activities” are particularly demanding due to physical factors, e.g., manual handling, repetitions, awkward and static postures and psychosocial risk factors [43].

In the transition between different age groups, we found that activities with no sick leave cases—‘’information and communication,” “financial and insurance activities” and “real estate activities” (Figure 1)—in younger workers tended to be less affected in all age groups (Figure 2, Figure 3 and Figure 4), with the exception of the number of sick leave cases for ‘’information and communication’’ and “financial and insurance activities” in the oldest group (Figure 4); however, these data are to be interpreted with caution due to the small number of employees (Table 2). Although there is an expected increase of musculoskeletal condition with age [4], this does not appear to be reflected in sick leave due to LBDs within the aforementioned activities. It would make sense to follow these activities to identify good practices that inhibit the growth of LBDs during workers aging and translate them into more critical activities.

In the oldest group (65+ age), we found some outstanding sick leave rates for LBDs in economic activities such as “administrative and support service activities,” “construction,” “agriculture, forestry and fishing,” and “public administration and defence; compulsory social security” (Figure 4), but we focused on SL rates related to LBDs only for economic activities in which more than 300 workers remain employed. Among such activities are manufacturing; wholesale and retail trade and repair of motor vehicles and motorcycles; professional, scientific, technical activities; education; and human health and social work activities (Table 2). The highest frequency due to LBDs was found for females in education and human health and social work activities. The reason is most likely a higher number of females compared to males who remain employed in these activities even after the age of 65 (Table 2) and in connection with the expected increase in musculoskeletal condition with age [4] and the duration of exposure to the working environment [2].

Comparisons to previous studies are difficult because this is the first nationwide data analysis on LBDs that used NACE Rev 2 classification. A large 2010 survey in the Netherlands identified the following groups to have an increase of incurring LBDs: male healthcare practitioners, female and younger healthcare support workers and female farming, fishing and forestry workers [44]. While some previous studies have indicated possible sex differences [23], our study shows that (a) the differences in LBD prevalence between sexes are negligible in the overall population and (b) large differences between the sexes may be seen in certain economic activities, which deserves further attention. Other research has been focusing on specific risk factors rather than individual economic activities. These include psychosocial, individual and biomechanical factors. For instance, load lifting, trunk bending and awkward postures have been associated with LBDs [45,46]. It can be speculated that performing such tasks heavily contributes to LBD incidence and severity in certain activities, as observed in this study (e.g., agriculture, forestry and fishing). Moreover, studies have linked LBDs to several psychosocial variables, such as job satisfaction, support in the workplace, job freedom and overtime work [47,48], which can be important in any economic activity. While risk factors for LBDs seem to be well documented, this study reveals which economic activities seem to be the most problematic and deserve further attention in terms of extensive research and practical implementation.

We must consider that individual NACE Rev2 economic activities cover different sub-activities that are not necessarily similar in type of work performed. Nevertheless, the results of our study can help decision-makers target the proper economic activity when developing strategies for reducing LBDs. The main aim of our study was not to provide a list of risk factors but to identify the most problematic economic activates that deserve immediate attention. Within the individual activities, it could be useful to identify the most exposed sub-activities in the future. However, we did not have available data to determine this in our study. 

It is known from the existing literature that a. higher prevalence of LBDs is associated with a physically demanding or sedentary type of work, especially if psychosocial risk factors are also present. The added value of our study is the magnitude of the data regarding the sick leave due to LBDs as we used nationwide data to analyze LBDs in various economic activities. The results of our study are useful in developing LBD control strategies in Slovenia and globally focusing on individual economic activities. 

### Limitations of the Study

The main limitation of the study is that we do not know the exact number of workers working at particular types of workplaces within an individual NACE Rev2 economic activity. For example, in “mining and quarrying,” we do not know the exact number of miners as this activity includes all the workers who work in mining and quarrying, including, for example, office workers in mining companies. 

Another limitation is that we do not know when the retirement of workers occurs. Worse sick leave rates in a particular activity for older workers could indicate that they retire later, thus having more LBDs than those already retired from other activities.

## 5. Conclusions

The purpose of the present study was to provide national data on sick leaves related to LBDs in order to identify economic activities that deserve the most attention in the future. A high prevalence of sick leave due to LBDs in Slovenia was present among young male workers in “mining and quarrying”. They are joint in the next age group (20.0–44.9 years) in the frequent occurrence of LBDs by men from “water supply; sewerage, waste management and remediation activities”. In the activities “administrative and support service activities,” a higher frequency in LBD-related sick leave is already manifested among younger employees and later followed by a longer average absence from work, especially in females. To better understand this phenomenon, further research is needed to identify biopsychological influences on the incidence of LBDs in “administrative and support service activities”.

Particular attention should be paid to “agriculture, forestry and fishing,” which showed a large average sick leave duration and thus probably a more demanding course of LBDs already in the case of young workers (20.0–44.9 years) of both genders. A similar phenomenon is seen in “construction” activities. 

We also advise the identification of good practices in activities in Slovenia that are less affected by LBDs: “information and communication,” “financial and insurance activities” and “real estate activities” and transferring them into more affected activities. According to gender, we have to pay attention to the longer average sick leave duration due to LBDs in younger females (20.0–44.9 years) and to the higher LBDs prevalence in females from “manufacturing” and “human health and social work activities”.

## Figures and Tables

**Table 1:** Active workers included in the study by gender, age and calendar year.

	Male	Female	All
2015	443,641	360,996	804,637
2016	446,863	370,346	817,209
2017	463,451	382,003	845,454
2018	478,148	394,624	872,772
2019	492,475	401,754	894,229

**Table 2:** The average size of the study population by age, gender and economic activities from 2015 to 2019.

Age gr.	15–19.9			20–44.9			45–64.9			65+		
Ec. Act.	M	F	TOTAL	M	F	TOTAL	M	F	TOTAL	M	F	TOTAL
A—agriculture, forestry and fishing	43	2	45	4629	2054	6683	5759	3474	9233	35	8	43
B—mining and quarrying	12	0	12	1238	178	1416	800	164	965	6	1	7
C—manufacturing	755	90	845	79,124	32,639	111,763	55,898	30,735	86,633	399	58	457
D—electricity, gas, steam, air cond. supply	5	0	5	3017	814	3831	3128	791	3920	49	6	55
E—water suppl; sewer., wst. manag., remed. act	17	1	18	3562	1014	4576	3816	1025	4841	21	2	23
F—construction	371	12	383	33,094	3211	36,305	22,196	2085	24,281	191	6	197
G—wholesale, retail; repair of mot. vehicles	237	110	347	33,306	36,835	70,140	19,618	20,971	40,589	252	87	339
H—transportation and storage	73	6	79	24,050	4898	28,948	18,268	4401	2269	236	9	245
I—accommodation and food ser. activities	122	114	236	9819	13,389	23,157	4401	7881	12,283	73	56	129
J—information and communication	8	2	10	12,572	5810	18,383	5477	2796	8272	76	21	97
K—financial and insurance activities	10	0	1	4483	7140	11,622	3385	6430	9816	27	12	39
L—real estate activities	5	1	6	1558	1063	2620	2367	1087	3454	40	9	49
M—professional, scientific, technical act	31	10	41	17,762	16,527	34,289	11,552	9365	20,917	449	144	593
N—administrative and support service act;	192	67	259	12,806	10,487	23,293	7418	6702	14,120	58	22	80
O—public admin, defense	11	2	13	11,872	13,080	24,952	11,286	13,528	24,815	169	69	239
P—education	15	18	33	8604	30,541	39,145	8151	27,914	36,065	305	125	430
Q—human health and social work; activities;	65	140	204	7304	27,087	34,391	4543	23,215	27,757	333	233	566
R—arts, entertainment and recreation	67	5	72	4429	4653	9082	3376	3169	6544	92	28	120
S—other service activities	26	93	119	2715	6938	9625	2033	3735	5766	63	59	121

M—male; F—female; A—agriculture, forestry and fishing; B—mining and quarrying; C—manufacturing; D—electricity, gas, steam, air cond. supply; E—water suppl; sewer., wst. manag., remed. act; F—construction; G—wholesale, retail; repair of mot. vehicles; H—transportation and storage; I—accommodation and food ser. activities; J—information and communication; K—financial and insurance activities; L—real estate activities; M—professional, scientific, technical act.; N—administrative and support service act; O—public admin, defense; compulsory soc. sec.; P—education; Q—human health and social work; activities; R—arts, entertainment and recreation; S—other service activities.

## Data Availability

All data is available in the paper and within the Supplementary Materials.

## Supplementary Materials

The following supporting information can be downloaded at: https://www.mdpi.com/article/10.3390/ijerph19010131/s1; Table S1. The overall results for days of absence, number of cases and average case duration because of sick leave due to low back disorders are presented separated by age, gender and NACE Rev 2 classification of economic activities.
