# Supplementary material for: The Prevalence and Severity of Sick Leave due to Low Back Disorders among Workers in Slovenia: Analysis of National Data across Gender, Age and Classification of Economic Activities
Source: Int J Environ Res Public Health. 2021 Dec 23;19(1):131. doi: 10.3390/ijerph19010131 (PMC8750890; doi:10.3390/ijerph19010131)
Supplement: Supplementary file 1 [file ijerph-19-00131-s001.zip › ijerph-1484903-supplementary.pdf]

International Journal of
Environmental Research and Public Health

MDPI

Supplementary

Table S1. The overall results for days of absence, number of cases and average case duration because of sick leave due to low back disorders are presented separated by age, gender and NACE Rev 2 classification of economic activities.

Age group	NACE REV Classification	Code	Days lost		N. cases		Duration	
			Male	Female	Male	Female	Male	Female
15-19.9 years	agriculture, forestry and fishing	A	0.0	0.0	0.0	0.0	/	/
20-44.9 years	agriculture, forestry and fishing	A	0.9	0.9	2.0	1.7	43.8	49.9
45-64.9 years	agriculture, forestry and fishing	A	2.1	2.6	2.4	3.1	86.3	82.4
>65 years	agriculture, forestry and fishing	A	4.6	1.0	1.1	2.4	401.0	41.0
15-19.9 years	mining and quarrying	B	2.7	0.0	6.5	0.0	42.0	/
20-44.9 years	mining and quarrying	B	2.6	0.8	8.2	3.4	31.2	23.4
45-64.9 years	mining and quarrying	B	5.0	0.3	8.5	3.5	58.8	9.1
>65 years	mining and quarrying	B	0.0	0.0	0.0	0.0	/	/
15-19.9 years	manufacturing	C	0.2	0.1	2.0	0.7	8.4	10.0
20-44.9 years	manufacturing	C	0.9	1.3	4.7	4.7	20.3	27.4
45-64.9 years	manufacturing	C	2.5	3.3	6.5	8.5	38.8	39.3
>65 years	manufacturing	C	1.2	0.0	2.7	0.3	42.9	5.0
15-19.9 years	electricity, gas, steam and air conditioning supply	D	0.0	/	0.0	/	/	/
20-44.9 years	electricity, gas, steam and air conditioning supply	D	0.6	0.6	4.2	3.4	13.8	17.5
45-64.9 years	electricity, gas, steam and air conditioning supply	D	1.6	1.9	5.8	6.0	27.3	32.6
>65 years	electricity, gas, steam and air conditioning supply	D	0.9	0.3	3.3	3.1	28.6	10.0
15-19.9 years	water supply; sewerage, waste management and remediation activities	E	0.1	0.0	1.2	0.0	8.0	/
20-44.9 years	water supply; sewerage, waste management and remediation activities	E	1.3	1.0	5.5	3.1	23.1	30.7
45-64.9 years	water supply; sewerage, waste management and remediation activities	E	2.8	1.9	7.2	6.3	38.2	29.4
>65 years	water supply; sewerage, waste management and remediation activities	E	1.3	0.0	3.7	0.0	35.5	/
15-19.9 years	construction	F	0.1	0.0	1.0	0.0	8.8	/
20-44.9 years	construction	F	0.9	0.5	2.9	1.4	30.8	37.2
45-64.9 years	construction	F	2.3	1.5	4.5	2.4	50.6	61.2
>65 years	construction	F	1.6	4.2	1.6	3.6	99.5	117.0
15-19.9 years	wholesale and retail trade; repair of motor vehicles and motorcycles	G	0.1	0.1	1.2	1.1	10.4	11.3
20-44.9 years	wholesale and retail trade; repair of motor vehicles and motorcycles	G	0.7	1.1	3.1	3.4	22.0	31.2
45-64.9 years	wholesale and retail trade; repair of motor vehicles and motorcycles	G	1.8	2.3	3.5	5.3	51.3	43.4
>65 years	wholesale and retail trade; repair of motor vehicles and motorcycles	G	0.7	0.3	1.0	0.7	68.8	42.0
15-19.9 years	transportation and storage	H	0.1	0.0	1.1	0.0	8.0	/
20-44.9 years	transportation and storage	H	0.7	0.8	3.3	3.0	22.0	26.2
45-64.9 years	transportation and storage	H	1.9	2.2	4.8	6.7	40.1	33.1
>65 years	transportation and storage	H	0.3	0.0	1.6	0.0	21.7	/
15-19.9 years	accommodation and food service activities	I	0.1	0.0	1.0	0.5	8.3	8.3
20-44.9 years	accommodation and food service activities	I	0.5	0.9	2.0	2.8	23.9	31.5

45-64.9 years	accommodation and food service activities	I	1.6	3.1	2.9	5.8	55.2	53.6
>65 years	accommodation and food service activities	I	0.2	0.2	0.5	0.4	35.5	60.0
15-19.9 years	information and communication	J	0.0	0.0	0.0	0.0	/	/
20-44.9 years	information and communication	J	0.3	0.4	1.6	2.2	15.2	15.8
45-64.9 years	information and communication	J	0.6	1.0	2.6	3.9	21.7	24.8
>65 years	information and communication	J	0.2	1.4	2.1	4.7	10.4	30.2
15-19.9 years	financial and insurance activities	K	0.0	0.0	0.0	0.0	/	/
20-44.9 years	financial and insurance activities	K	0.3	0.5	2.5	3.2	13.8	16.7
45-64.9 years	financial and insurance activities	K	1.1	1.1	4.0	5.3	27.1	21.5
>65 years	financial and insurance activities	K	0.3	0.0	2.2	0.0	15.3	/
15-19.9 years	real estate activities	L	0.0	0.0	0.0	0.0	/	/
20-44.9 years	real estate activities	L	0.5	0.4	3.0	2.2	15.8	19.5
45-64.9 years	real estate activities	L	1.3	1.0	4.8	3.6	26.6	27.9
>65 years	real estate activities	L	0.0	0.0	0.5	0.0	7.0	/
15-19.9 years	professional, scientific and technical activities	M	0.1	0.0	1.3	0.0	4.5	/
20-44.9 years	professional, scientific and technical activities	M	0.3	0.3	1.5	1.3	22.0	24.3
45-64.9 years	professional, scientific and technical activities	M	0.7	1.0	1.8	2.2	38.4	44.3
>65 years	professional, scientific and technical activities	M	0.1	1.2	0.3	0.8	27.6	139.0
15-19.9 years	administrative and support service activities	N	0.4	0.3	4.4	2.4	8.8	13.4
20-44.9 years	administrative and support service activities	N	1.0	1.0	5.5	4.3	18.5	24.5
45-64.9 years	administrative and support service activities	N	2.4	3.5	6.4	7.5	37.8	46.1
>65 years	administrative and support service activities	N	5.1	15.2	3.8	3.7	134.7	411.5
15-19.9 years	public administration and defence; compulsory social security	O	1.8	0.0	5.5	0.0	33.3	/
20-44.9 years	public administration and defence; compulsory social security	O	1.4	1.2	7.1	5.6	19.0	20.7
45-64.9 years	public administration and defence; compulsory social security	O	2.5	2.2	6.9	8.5	35.9	26.1
>65 years	public administration and defence; compulsory social security	O	1.1	0.8	5.8	5.2	19.8	14.4
15-19.9 years	education	P	0.1	0.0	1.3	0.0	8.0	/
20-44.9 years	education	P	0.3	0.6	1.9	3.2	16.0	18.6
45-64.9 years	education	P	0.7	1.8	2.7	6.4	24.5	27.6
>65 years	education	P	0.6	0.8	1.6	3.7	36.1	20.6
15-19.9 years	human health and social work activities	Q	0.0	0.1	0.9	0.9	4.0	10.0
20-44.9 years	human health and social work activities	Q	0.8	1.2	3.9	4.5	20.5	26.3
45-64.9 years	human health and social work activities	Q	1.7	2.9	5.4	8.4	30.9	35.0
>65 years	human health and social work activities	Q	1.2	0.7	1.6	2.7	79.6	25.2
15-19.9 years	arts, entertainment and recreation	R	0.0	0.0	0.3	0.0	5.0	/
20-44.9 years	arts, entertainment and recreation	R	0.5	0.5	1.7	1.7	27.7	26.4
45-64.9 years	arts, entertainment and recreation	R	1.0	1.7	3.1	4.6	33.0	37.2
>65 years	arts, entertainment and recreation	R	1.8	0.2	1.5	2.9	117.1	6.8
15-19.9 years	other service activities	S	0.1	0.0	0.8	0.6	11.0	6.7
20-44.9 years	other service activities	S	0.3	0.5	1.4	1.6	21.4	34.0

45-64.9 years	other service activities	S	0.9	1.6	1.6	2.4	56.5	65.2
>65 years	other service activities	S	0.4	0.6	1.3	1.0	30.5	57.7
